# Detecting a single molecule using a micropore-nanopore hybrid chip

**DOI:** 10.1186/1556-276X-8-498

**Published:** 2013-11-21

**Authors:** Lei Liu, Lizhong Zhu, Zhonghua Ni, Yunfei Chen

**Affiliations:** 1 Jiangsu Key Laboratory for Design and Manufacture of Micro-Nano Biomedical Instruments, School of Mechanics, Southeast University, Nanjing 210096, People's Republic of China; 2 Suzhou Key Laboratory for Design and Manufacture of Micro-Nano Biomedical Instruments, Suzhou Research Institute of Southeast University, Suzhou 215123, People's Republic of China

**Keywords:** Nanopore, Biosensing, Biomolecule

## Abstract

Nanopore-based DNA sequencing and biomolecule sensing have attracted more and more attention. In this work, novel sensing devices were built on the basis of the chips containing nanopore arrays in polycarbonate (PC) membranes and micropores in Si_3_N_4_ films. Using the integrated chips, the transmembrane ionic current induced by biomolecule's translocation was recorded and analyzed, which suggested that the detected current did not change linearly as commonly expected with increasing biomolecule concentration. On the other hand, detailed translocation information (such as translocation gesture) was also extracted from the discrete current blockages in basic current curves. These results indicated that the nanofluidic device based on the chips integrated by micropores and nanopores possessed comparative potentials in biomolecule sensing.

## Background

Since voltage-driven biomolecule translocation through nanopores was first reported by Kasianowicz et al. in 1996 [1], nanopores in solid films have become an effective tool for bio-analysis [2-4]. Nowadays, more and more theoretical and experimental studies aiming to design nanopore-based sensing device have been carried out, and most of them are at the forefront of life sciences, chemistry, material sciences, and biophysics. For example, nanopore plays an important role in low-cost and rapid DNA sequencing, which is expected to have major impact on bio-analysis and to give fundamental understanding of nanoscale interactions down to single-molecule level. The mechanism of nanopore-based biomolecule sensing or DNA sequencing can be simply depicted as follows: analyte in electrolyte solution is driven through a nanopore by applied electric field, yielding a characteristic change in background ionic current due to its translocation. According to the existed work, analyte with its dimensions comparable to the size of nanopore is quite advantageous to obtain signals with better quality. The concentration information of analyte can be obtained by comparing the frequencies of translocation events, while the structural information of analyte can be acquired by analyzing the magnitude, duration, and shape of the current blockages. In addition, pore geometry, pore size, flow direction, and other factors also have influences on the detected current signals. On the other hand, theoretical studies in this area have attempted to give a fundamental understanding of the translocation, which are expected to obtain deeper comprehensions for the relevant existing experiments [5]. Fabrication of smart nanopore-based device together with the sensitive collection and accurate analysis of current signals is regarded as a key issue in nanopore-based analysis and DNA sequencing.

Generally speaking, natural pores at nanometer scale (such as alpha-hemolysin) in biomembranes and artificial pores at nanometer scale in solid films are two major types of nanopores used in DNA sequencing and biomolecule sensing. In this area, Bayley and Cremer [6], and Bayley and Jayasinghe [7] have performed fundamental studies on alpha-hemolysin. On the basis of these pioneer efforts, other excellent research work on protein-based nanopore has been carried out [8,9]. In recent years, the developments of artificial nanopores have become faster and faster with the rapid developments of nanoscience and nanotechnology. Novel fabricating methods, such as ion beams and electron beams [10-12], have been gradually used to manufacture artificial nanopore in thin solid materials (including silicon nitride [13-17], graphene [18-21], and silicon oxide [22,23]) for sequencing or bio-analysis usage. These progresses are of great importance for nanopore-based sensing devices because of their great potentials in combination with developed MEMS technology. In addition, the group of Harrell et al. and other groups have utilized track etching method to prepare conically-shaped single nanopore in polymer membranes (such as polycarbonate, poly(ethylene terephthalate), polypropylene, poly-(vinylidene fluoride), and polyimide), which provides other possible choice for nanopore-based sensing device [24-27].

In this work, novel sensing devices were fabricated on the basis of nanopore arrays in polycarbonate (PC) membranes and micropores in Si-Si_3_N_4_ films, and related translocation properties of single molecule were investigated using these devices.

## Methods

### Experimental device and reagent

PC membranes containing nanopore (pore diameter 50 nm, pore density six pores per μm^2^, membrane thickness 6 to 11 μm) arrays were purchased from Whatman, Inc. (Shanghai, China), and hydrophilic treatments were carried out before usage. Ultrapure water (18.25 MΩ · cm) was used for the preparation and rinsing. Goat antibody to human immunoglobulin G (IgG) and *λ*-DNA (48 kB, 310 ng/mL) obtained from Nanjing Boquan Technology Co., Ltd. (Jiangsu, China) were used as analytes in the experiments. Potassium chloride (KCl) was commercially available and at analytical grade.

A test device containing separated liquid cells linked by nanopore chip (sealed by PDMS) was integrated to measure the ionic current. At room temperature (25°C ± 2°C), KCl solution (pH = 7.48) was added to both feed cell and permeation cell, and the analytes were dissolved in the reservoir. An electric field was applied to both sides of the nanopore chip, and the transmembrane current was recorded using a Keithley2000 61/2-digital multimeter (Cleveland, OH, USA) or HEKA EPC-10 patch clamp (Bellmore, NY, USA). The nanopores were characterized using a MFP-3D-SA atomic force microscope produced by Asylum Research (Goleta, CA, USA). The micropores in the Si_3_N_4_ film was fabricated and characterized using Helios NanoLab 600i dual beam (Hillsboro, OR, USA).

### Fabrication of nanopore-based device

**Figure 1:**
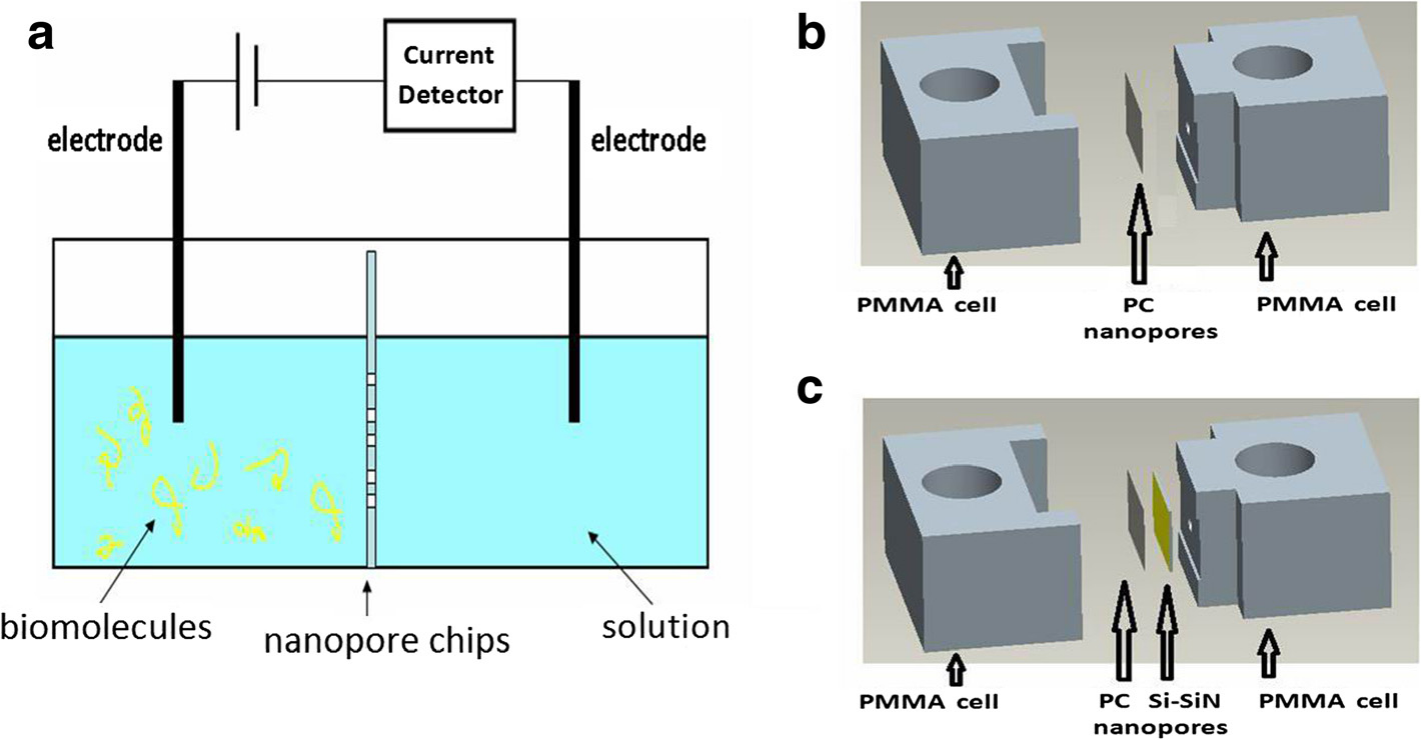
**The sensing device. (a)** The prototype nanofluidic device based on integrated micro-nano pore for biosensing. The left cell in which the biomolecules are added is the feed cell, and the right cell is the permeation cell. **(b)** The designed sensing devices were built using only PC nanopore membrane for ionic current detection. **(c)** The designed sensing devices containing PC nanopore membrane integrated with Si_3_N_4_-Si hybrid micropore structure for biomolecule sensing.

The scheme of the fabricated nanofluidic device for biosensing is shown in Figure 1a: two separated liquid cells filled with KCl solution are linked by nanopore chip; certain voltage is applied along the axial direction of the nanopore, which results in background ion current. The analytes in the electrolytic solution are electrophoretically driven to pass through the nanopore, and the translocation events can be marked by the changes in the background currents. In our work, two kinds of chips, the chip containing micropore in Si_3_N_4_-Si film covered by PC nanopores arrays (here ‘nanopores arrays’ means many nanopores which are distributed in a two-dimensional area, or many parallel nanochannels which are distributed in a three-dimensional area) and the chip containing only PC nanopore arrays (shown in Figure 1b, c, respectively), were employed for single-molecule sensing.

**Figure 2:**
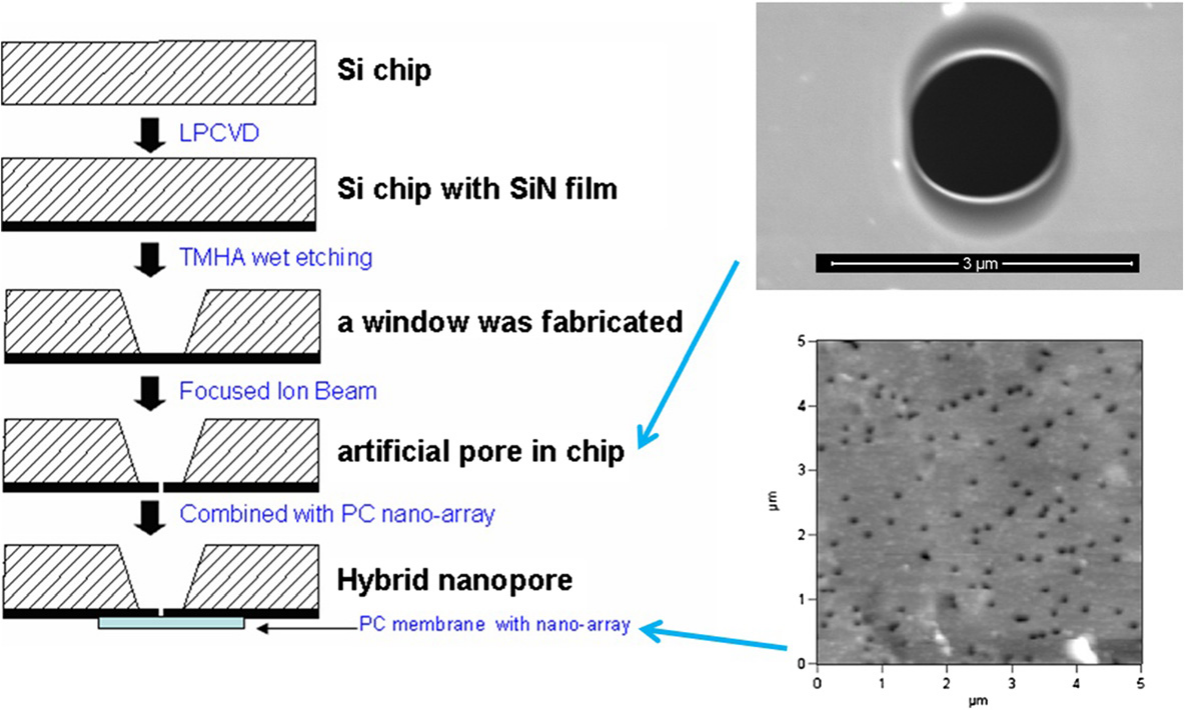
**Illustration of the integration process of micropore.** (1) Si_3_N_4_film on one side of the Si chip was obtained by LPCVD method. (2) A window on the top of chip at Si side was fabricated by wet etching. (3) Artificial micropores on the Si_3_N_4_film were fabricated by FIB. (4) PC membrane was covered on the Si_3_N_4_pore and sealed using PDMS. The inset in the top right corner is the SEM image of Si_3_N_4_ micropore; the inset in the lower right corner is an AFM image of the PC nanopore arrays.

The micropores in the Si_3_N_4_ film were fabricated and integrated with PC nanopore membranes according to the following steps (Figure 2): (1) a Si_3_N_4_ film (thickness about 100 nm) on one side of the Si chip (5 mm × 5 mm) was obtained by low-pressure chemical vapor deposition (LPCVD) method, (2) a window on top of the chip at the Si side was fabricated by wet etching using tetramethylammonium hydroxide (TMHA), (3) the artificial micropores on the Si_3_N_4_ film were fabricated and characterized using focused ion beam (FIB) and scanning electron microscope (SEM), and (4) the Si_3_N_4_ micropore was covered by PC membrane containing nanopores (pore size 50 nm) and sealed using polydimethylsiloxane (PDMS). After these steps, hybrid chips were obtained for further nanofluidic device integration and biosensing.

## Results and discussion

### Fabrication of nanopore-based device

**Figure 3:**
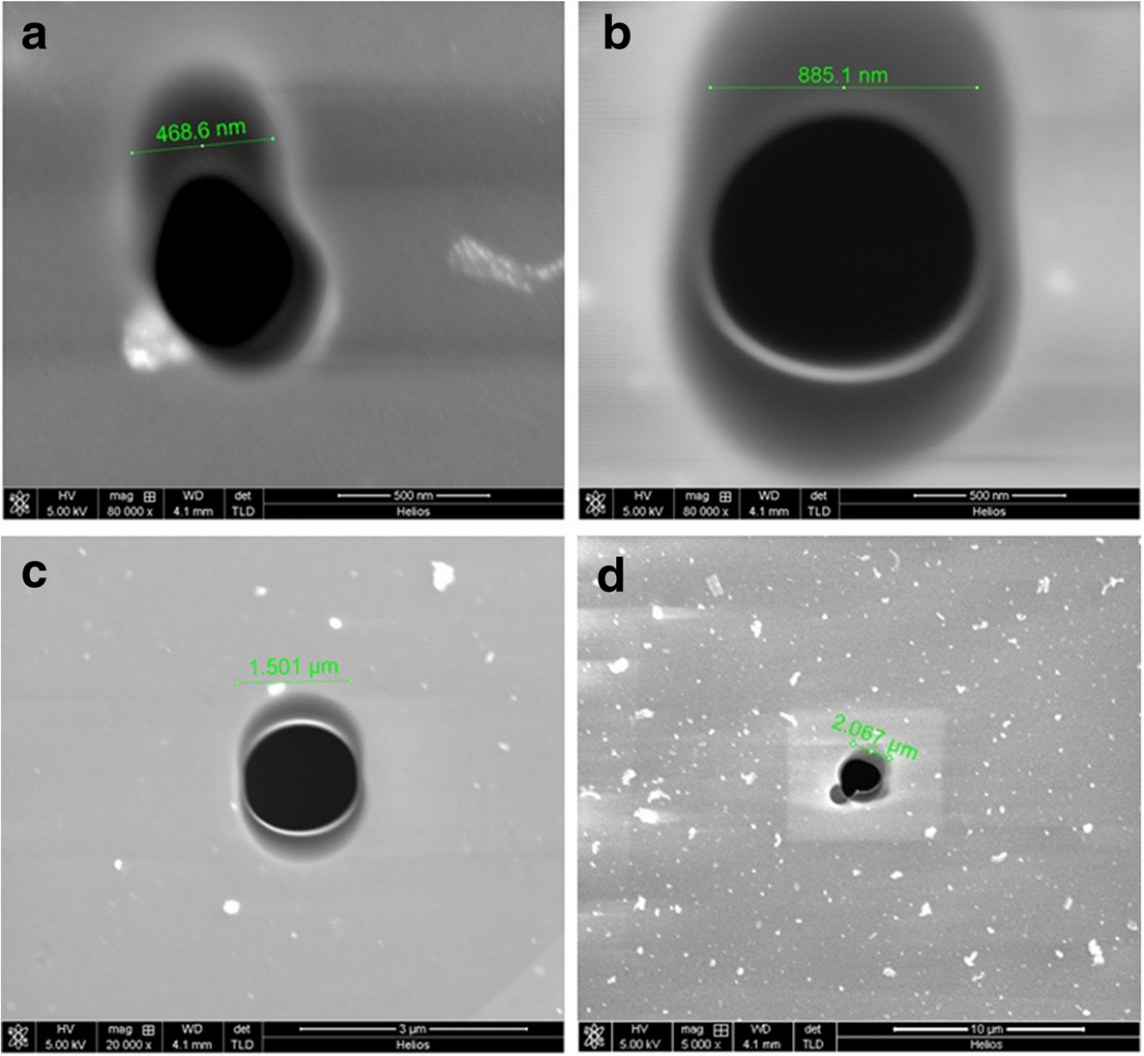
**SEM images of the Si**_**3**_**N**_**4 **_**micropores with different diameters in Si-Si**_**3**_**N**_**4 **_**hybrid structures. (a)** 0.47 μm, **(b)** 0.88 μm, **(c)** 1.5 μm and **(d)** 2.0 μm.

In our experiment, PC ultrafiltration membranes are employed as nanopore arrays, whose size and distribution are characterized using an atomic force microscope. The AFM image shown in Figure 2 gives the size and distribution information of the nanopore arrays: their pore size is 50 nm or so, and they are distributed randomly in the membrane. The micropores in the Si_3_N_4_ films were fabricated using focused Ga^+^ beam. Obviously, the size and shape of the pore are mainly determined by the energy of the Ga^+^ beam and irradiation time. Generally speaking, greater beam energy corresponds to rather faster processing speed. Meanwhile, the irradiation time should exceed a threshold value to guarantee the film being penetrated. In a certain range, the pore size will gradually increase with increasing irradiation time. By controlling the proper beam energy and irradiation time, four Si_3_N_4_ pores with sizes of 0.47, 0.88, 1.5, and 2.0 μm are obtained, as shown in Figure 3. If these pores are regarded as ideal round, the calculated pore areas are 0.16, 0.61, 1.77, and 3.14 μm^2^, respectively. Considering the calculated pore areas and the distribution status of the nanopore, theoretical amounts of ‘uncovered’ nanopores are 0.96, 3.66, 9.84, and 18.84, respectively. At the same time, the total amounts of the uncovered nanopores are also influenced by the heterogeneity of their distribution and other related factors (for example, it is difficult to control PDMS to exactly arrive at the edge of the micropore. Less mobility of PDMS at the beginning of the solidification may make it exceed the edge of the micropore, which will result in the decrease of effective pore size or even pore closing). According to our experimental experience, if the size of Si_3_N_4_ pore is less than 1 μm, it is difficult to guarantee the success of further ionic current detection. In our experiment, micropores with sizes of 1.5 and 2.0 μm have been employed.

### Ionic currents induced by biomolecule translocation

The sensing device based on PC membranes containing nanopore arrays was used to detect the ionic currents modulated by the biomolecule's translocation. KCl solutions of 0.001, 0.01, and 0.1 mol/L were employed as electrolytes, and IgG was used as analyte.

**Figure 4:**
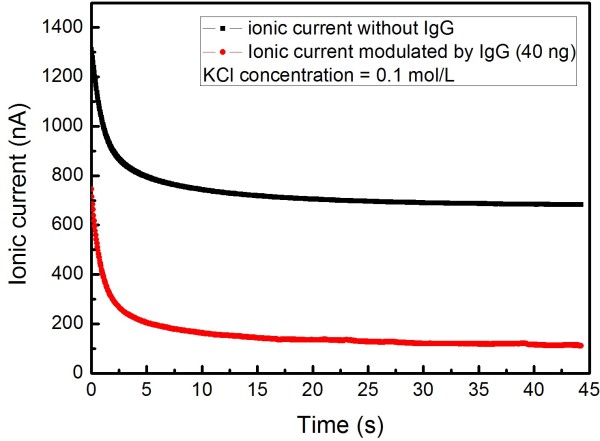
**Ionic current modulated by IgG translocation through nanopore arrays.** The black line and red line stands for the detected background ionic current curve and modulated ionic current curve, respectively (the driven voltage is 1.0 V, and KCl concentration is 0.1 mol/L).

**Figure 5:**
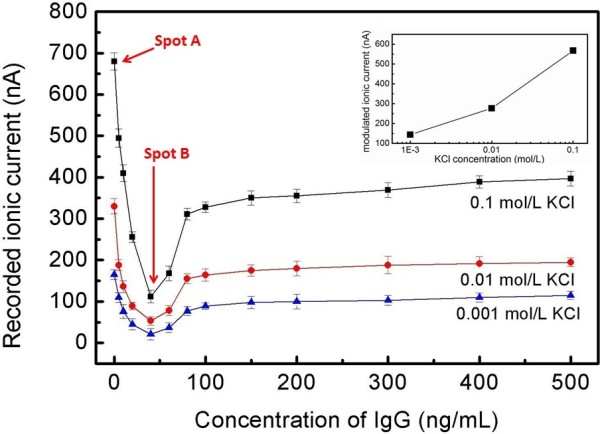
**The recorded ionic current versus the variation of IgG concentration in 0.1 mol**/**L KCl solution.** The applied voltage is 1 V. The diameter of the nanopore arrays is 50 nm. The inset in the top right corner shows the differences between the background currents and the recorded currents at 40 ng/mL of IgG for different KCl concentrations.

As mentioned above, there are many, many nanopores in the PC nanopore membrane (pore density six pores per μm^2^). If only the PC nanopore membrane is used, the effective nanopore number is about 106 to 107, which is a very big amount. From a probabilistic perspective, a lot of IgG molecules will pass through the nanopore arrays simultaneously. In this case, it is almost impossible to find occasional current blockade in the background current curve. On the contrary, large number of biological molecule translocations result in the statistical superposition effect in the modulation in the base current, which is embodied in the decrease in the background current. Figures 4 and 5 show the ionic current changes induced by IgG translocation only through nanopore arrays. In Figure 4, the black and red lines stand for the detected background ionic current curve and the modulated ionic current curve, respectively (the driven voltage is 1.0 V, and KCl concentration is 0.1 mol/L). The background ionic current value is stable at 680 nA, which corresponds to *spot A* in Figure 5. When the biomolecules are added, their translocations result in the decline of the current; so, the modulated ionic current value is stable at 110 nA, which corresponds to *spot B* in Figure 5.

Figure 5 shows the detected current changing, with IgG concentration increasing at the driven voltage of 1.0 V. The differences between the background currents and the modulated currents versus KCl concentrations (IgG concentration is 40 ng/mL) are plotted, as shown in the inset of Figure 5, which reflects the influence on the ionic current caused by the concentration of electrolyte solution. If KCl concentration continues to increase, the ion density in the solution becomes higher and higher. Then, the lost amounts in K^+^ and Cl^−^ due to the physical place-holding effect are rather bigger. On the other hand, the obtained results about the current changing tendency with IgG concentration indicate that the detected ionic current decreases with IgG concentration increase when it is lower than 40 ng/mL. Obviously, the entry of the IgG molecules results in the partial occupations of nanopore arrays, which prevents K^+^ and Cl^−^ from passing through the PC membrane. Within a certain concentration, the translocation probability of IgG increases with its increasing concentration. As we have known, the volume of IgG is much larger than that of K^+^ or Cl^−^, so the charge density is rather lower in the occupied channel space, which results in the decrease in the detected ionic current.

However, the ion current does not continue to drop with increasing IgG concentration as expected; on the contrary, the ionic current increases with the increasing IgG concentration when it is higher than 40 ng/mL, and then it tends to be stable with the concentration continuing to increase, as shown in Figure 5. An example will help us understand this phenomenon: imagine a big room with a door; the maximum allowable value of the entering people in unit time is *N*. When the number of people who need to enter the room in unit time is lower than *N*, the value of the entering people in unit time will increase with the increasing number of people who need to enter. When the number of people who need to enter the room in unit time is larger than *N*, the actual value of the entering people will equal to or even be lesser than *N* (especially for disordered conditions). Similarly, when IgG concentration is higher than the threshold value, the number of passing molecules will remain or decrease. The physical place-holding effect is weakened, which can result in the increase of ionic current.

### Single-biomolecule sensing

Only an overall decline in the background current can be observed using PC membranes. In order to find the changes in the background current curve induced by a single biomolecule's translocation, the Si_3_N_4_ micropore is employed, and it is covered by the PC membrane containing nanopore arrays, which will significantly decrease the effective nanopore numbers. The effective areas of the two Si_3_N_4_ micropores used in our work are 1.77 μm^2^ (chip 1) and 3.14 μm^2^ (chip 2), which can decrease the effective nanopore number from 10^6^ and 10^7^ to 10 and 19, respectively. They are integrated into the nanofluidic device for DNA sensing, and the ionic current was recorded by patch clamp. In these cases, the probabilities of the simultaneous translocation events decreased dramatically. So, it is possible to obtain discrete ionic drops or blockades in the detected ionic curves during biomolecules' translocations, which can provide more information for the translocation.

**Figure 6:**
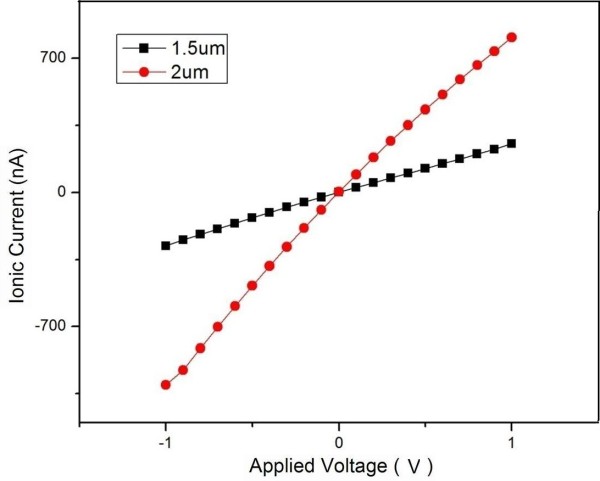
**The characteristic *I-V *curves for the integrated micro-nano pore in 0.75 mol/L KCl solution.** The sizes of the Si_3_N_4_ pores are 1.5 and 2.0 μm.

**Figure 7:**
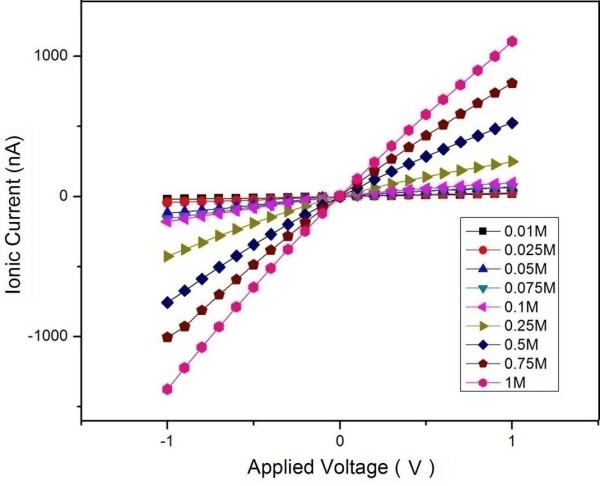
**The characteristic *I-V *curves for the integrated micro-nano pore in different KCl solutions.** The size of the Si_3_N_4_pore is 2.0 μm; the KCl solutions are from 0.01 to 1.0 mol**/**L.

Figure 6 shows the characteristic *I*-*V* curves obtained using chip 1 and chip 2, respectively. The theoretical amounts of the effective nanopores in chip 1 and chip 2 are 10 and 19, respectively. The results indicate that chip 2 processes bigger ionic conductance compared with chip 1. Obviously, more effective nanopores correspond to more permeated areas, which can allow more ion translocations and result in bigger ionic currents, supposing that other conditions (such as concentration of electrolyte solution, applied voltage, pH value, and temperature) are not changed. For one integrated chip, higher concentration of KCl solution results in bigger ionic current if the other conditions are not changed, as shown in Figure 7. This is due to the increase of ion in the unit solution volume.

**Figure 8:**
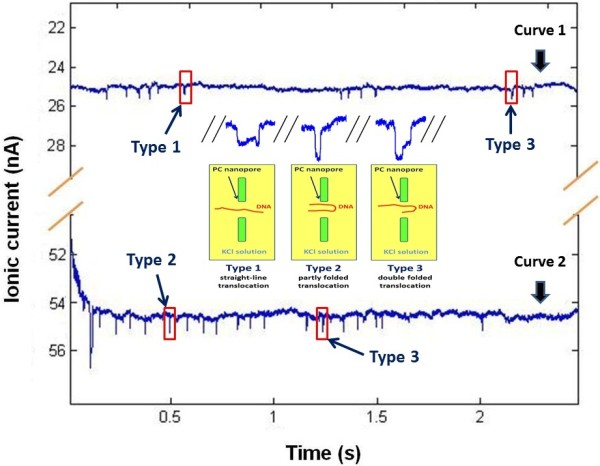
**Simultaneous ionic current measurements of DNA translocation based on integrated micro**-**nanopore chip.** The applied voltage is 1 V, and the concentration of KCl solution is 0.01 mol/L. Curve 1 is obtained using chip 1; curve 2 is obtained using chip 2.

The top current curve and bottom current curve in Figure 8 are obtained from chip 1 and chip 2, respectively, which show some discrete blockages in the background current induced by DNA translocation. The base lines of the detected ionic currents are stable at 26 nA for chip 1 and 54 nA for chip 2. The blockage appears in the base current curves randomly, which correspond to the different translocation event. Because of more effective nanopore numbers in chip 2, the translocation frequency in this chip is rather higher than that in the case using chip 1. For both cases, the amplitudes of blockades vary from 0.5 to 1.0 nA. The directional movement of DNA temporarily changes the original ionic current, which is generated by the directional movements of K^+^ and Cl^−^. When the DNA molecules are added into the solution, they will be driven to pass through the integrated chip by electric field force. First, the physical place-holding effect caused by DNA translocation changes the ionic current simultaneously and results in blockages in current curve. Some positions in the nanopores are partially occupied by DNA, which prevents certain amounts of K^+^ and Cl^−^ from translocating. This decreases the ionic current which is generated by K^+^ and Cl^−^. On the other hand, when DNA passes through the nanopore, its surface charge also contributes to the increase of the detected ionic current. The final current changes are determined by the comprehensive effect of the above factors. If the electrolyte concentration is quite higher (ion density in solution is higher), the lost amounts of ions due to the physical place-holding effect will be quite bigger. At the same time, the surface charge of DNA does not change when the pH value remains. If the current drop caused by the physical place-holding effect is bigger than the current increase caused by the DNA surface charge, it will result in a final decrease blockage in the base current; on the contrary, if the concentration of electrolyte is quite lower and the current drop caused by physical place-holding effect is smaller than the current increase caused by DNA surface charge, it will result in a final increase blockage in the base current, as shown in Figure 8.

On the other hand, the blockages in the base current curve can also provide detailed information of DNA translocation. The inset in the center of Figure 8 shows different magnified blockages, which stand for the three main types of DNA translocation gestures discriminated from varieties of translocation data, as following: (1) straight-line translocation: in this case, the decrease of ionic current at the blockages is smaller, while the duration time is rather bigger; (2) double-folded translocation: in this case, the decrease of the ionic current at the blockades is about twice that in case 1, but the duration time is only a half; (3) partly-folded translocation: in this case, the decrease of the ionic current at blockades and the duration time are both between case 1 and case 2, and the shapes and durations of the blockades change variously because of the different gestures in DNA translocation. Comparing the two curves in Figure 8, the amounts of the effective nanopore numbers can be modulated by adjusting the size of the Si_3_N_4_ micropore, which can change the frequency of the current drop signals in the ionic current curve.

## Conclusions

In summary, the transporting properties and detailed translocation information of biomolecules are investigated using an integrated device based on nanopore arrays in PC membranes and micropore in silicon nitride films. The amounts of effective nanopore numbers can be modulated by adjusting the size of Si_3_N_4_ micropore, which can change the frequency of signals in ionic current curve. It is believed that the nanofluidic device based on integrated micropore-nanopore chips possessed comparative potentials in biosensing applications.

## Competing interests

The authors declare that they have no competing interests.

## Authors' contributions

LL carried out the experimental design, part of the experimental work and data analysis, and drafted the manuscript. LZ carried out part of the experimental work. ZN and YC participated in the result discussions. All authors read and approved the final manuscript.

## Authors' information

LL is an associate professor at the Southeast University, PR China. LZ is an undergraduate student at the same university. ZN and YC are professors at the Southeast University, PR China.
